# Evaluation of the Diagnostic Accuracy of *CareStart* G6PD Deficiency Rapid Diagnostic Test (RDT) in a Malaria Endemic Area in Ghana, Africa

**DOI:** 10.1371/journal.pone.0125796

**Published:** 2015-04-17

**Authors:** Dennis Adu-Gyasi, Kwaku Poku Asante, Sam Newton, David Dosoo, Sabastina Amoako, George Adjei, Nicholas Amoako, Love Ankrah, Samuel Kofi Tchum, Emmanuel Mahama, Veronica Agyemang, Kingsley Kayan, Seth Owusu-Agyei

**Affiliations:** Kintampo Health Research Centre, P O Box 200, Kintampo, Brong Ahafo, Ghana; Centro de Pesquisa Rene Rachou/Fundação Oswaldo Cruz (Fiocruz-Minas), BRAZIL

## Abstract

**Background:**

Glucose-6-phosphate dehydrogenase (G6PD) deficiency is the most widespread enzyme defect that can result in red cell breakdown under oxidative stress when exposed to certain medicines including antimalarials. We evaluated the diagnostic accuracy of *CareStart* G6PD deficiency Rapid Diagnostic Test (RDT) as a point-of-care tool for screening G6PD deficiency.

**Methods:**

A cross-sectional study was conducted among 206 randomly selected and consented participants from a group with known G6PD deficiency status between February 2013 and June 2013. A maximum of 1.6ml of capillary blood samples were used for G6PD deficiency screening using *CareStart* G6PD RDT and Trinity qualitative with Trinity quantitative methods as the “gold standard”. Samples were also screened for the presence of malaria parasites. Data entry and analysis were done using Microsoft Access 2010 and Stata Software version 12. Kintampo Health Research Centre Institutional Ethics Committee granted ethical approval.

**Results:**

The sensitivity (SE) and specificity (SP) of *CareStart* G6PD deficiency RDT was 100% and 72.1% compared to Trinity quantitative method respectively and was 98.9% and 96.2% compared to Trinity qualitative method. Malaria infection status had no significant (P=0.199) change on the performance of the G6PD RDT test kit compared to the “gold standard”.

**Conclusions:**

The outcome of this study suggests that the diagnostic performance of the *CareStart* G6PD deficiency RDT kit was high and it is acceptable at determining the G6PD deficiency status in a high malaria endemic area in Ghana. The RDT kit presents as an attractive tool for point-of-care G6PD deficiency for rapid testing in areas with high temperatures and less expertise. The *CareStart* G6PD deficiency RDT kit could be used to screen malaria patients before administration of the fixed dose primaquine with artemisinin-based combination therapy.

## Background

Glucose-6-phosphate dehydrogenase (G6PD) deficiency is the largest and most widespread enzyme defect affecting approximately 400 million persons worldwide. In malaria endemic areas, the prevalence of G6PD ranges from 5.0% to 23.8% [1]. It is a sex-linked genetic defect occurring on the X-chromosome. The global distribution of G6PD-deficient variants resembles the geographical distribution of malaria and this supports the thinking that G6PD deficiency confers some protection against malaria [2]. A number of G6PD deficient variants have been reported in sub-Saharan Africa. Although there are over 400 G6PD variants worldwide, in sub-Saharan Africa three variants, though not exclusive, occur; G6PD*B, G6PD*A and G6PD*A-. G6PD*B is the wild type and the most common variant in Africa and worldwide. G6PD*A has a normal variant with about 90% of the G6PD*B enzyme activity [1, 3], while G6PD*A- is a deficient variant with about 8–20% of the G6PD*B enzyme activity [1]. Deficiency can result in destruction of red cells and depending on the G6PD variant present, haemolysis maybe mild, moderate, or severe [4, 5].

Malaria, the most common parasitic disease which is estimated to infect 3.2 billion people in 97 countries and territories [6] is caused by infectious disease agents *Plasmodium Vivax (Pv)*, *P*. *falciparum* (*Pf*), *P*. *ovale (Po)*, *P*. *malariae (Pm)*, and the recently emerging *P*. *knowlesi (Pk)*. The burden of malaria is heaviest in the WHO African Region, where an estimated 90% of all malaria deaths occur, and in children aged under 5 years, who account for 78% of all deaths [6].

In Ghana, the entire nation is at the risk of malaria infection, particularly in the middle belt of Ghana where transmission occur all year round with about 22.8% of malaria parasitaemia of which 98.1% is as a result of *Plasmodium falciparum* infection [7, 8]. The country has been involved in the malaria control program conducted by the WHO since early 2000. A very large number of cases are attributable to *Pf* infection [7, 8] with no documented records of infections due to *Pv [9]* as happens in the eastern part of Africa. The prevalence of G6PD deficiency in Ghana has been reported as 6.5% [1]. In the middle belt with a population mainly of Akan and Mo ethnic background [10], the G6PD prevalence has been reported as 6.8% and 19% [1, 11]. All reported deficient G6PD*A- genotypes were A376G/G202A and there were no instances of A542T, G680T or T968C in the study that looked at the G6PD genotype [1]. The absence of no record of *Pv* relapsing malaria parasite infection [9] might have influenced the absence of the use of primaquine from its Standard Treatment Guidelines for malaria [12]. It is of interest to note that with the variant of G6PD deficient genotype (G6PD*A-) prevalent in Ghana, there have been no report of any drug implicated to cause G6PD-related hemolysis except for clinical trials of chlorproguanil-dapsone artesunate (CDA) [5]. Being aware of primaquine inducing acute haemolytic anaemia, participants with G6PD deficiency were exempted from taking part in a study that used primaquine to radically clear malaria parasites [13].

People with G6PD deficiency are mostly asymptomatic but exposure to oxidant drugs, such as the anti-malarial drug primaquine, may induce hemolysis. The WHO recommended the use of a single dose of primaquine to artemisinin-based combination therapy (ACT) as a component of pre-elimination and elimination programmes provided the G6PD deficiency status of patient is determined [14, 15].

Due to the seldom availability of G6PD testing in the field the implementation of the recommendation was limited. This challenge led to a review of the recommendation to a single dose of 0.25 mg base/kg primaquine, a dose considered unlikely to cause serious toxicity in subjects with any of the G6PD variants [14]. Despite the revised recommendation by WHO, it is still important to make G6PD testing available in the field to eliminate any haemolytic effect at any dose of primaquine especially in Ghana where G6PD A- variant is common [1].

Phenotypic tests (being it quantitative or qualitative), unlike genetic techniques, to determine G6PD activity status are the most widely used and are the best indicators of G6PD function levels. Some of these testing products are suitable for point-of-care use in clinics because they are rapid and straightforward [2]. However, most of these tests require relatively sophisticated laboratory capacities equipped with electricity or specific laboratory equipment to regulate temperature [16] and also larger volumes of blood.

A rapid and inexpensive methods to determine the G6PD status of all persons is desirable when one is considering use of drugs contraindicated in patients with G6PD deficiency. It is especially important and particularly needed in sub-Saharan Africa where G6PD deficiency is high, malaria is endemic and use of oxidative malarial drugs such as primaquine is expectedly high. One of such phenotypic biochemical tools; the *CareStart* G6PD RDT kit is developed to overcome some of the challenges faced when one uses the existing routine G6PD screening methods before patient management. The Kit is rapid, works in wide range of temperature [16], uses small amounts of blood (*Manufacturer’s manual)* and not costly compared to other commercially available G6PD test kits [17].

To the best of our knowledge, *CareStart* G6PD RDT kit has not been evaluated in a malarial endemic area in an African setting as compared to what has been done largely by others elsewhere. The use of the device for point-of-care testing has been described as feasible, not labour intensive, require less training, not time involving, no special additional equipment and storage conditions required and has the ability to detect G6PD deficient individuals [2, 16–20]. Majority of these evaluation studies which occurred outside Africa used venous blood samples except Bancone et al, 2015 [20] which employed capillary blood collection from sterile finger prick to assess the performance of the RDT kit.

We evaluated the diagnostic accuracy of the *CareStart* G6PD deficiency RDT kit using capillary blood in a malarial endemic area in Ghana, Africa where G6PD deficiency is high and the need for RDT to rapidly screen for G6PD deficiency is urgent. This is necessary to make a tool available at point-of-care when Ghana adopts the recommendation of WHO to use fixed dose of primaquine for malaria treatment and elimination programme.

## Methods

### Ethical statement

Ethical approval of the study procedures and consultations of records of potential participants with known G6PD status to recruit was granted by the Kintampo Health Research Centre Institutional Ethics Committee (KHRC-IEC). The objectives and procedures of the study were carefully explained to all potential participants to seek their written/thumb printed informed consent to participate. Care-takers gave written informed consent on behalf of their minor participants and also on behalf of their children who were between ages 12 and 17, who additionally gave their written/thumb printed informed assent to be part of the study. Participants were informed they were being contacted because of their previous involvement with the Kintampo Health Research Centre for studies that involved G6PD testing which they gave their written informed consent to be contacted anytime needful. Participants’ identity and records were anonymized prior to analysis.

### Study design

We adopted a cross-sectional study design to evaluate the performance of the *CareStart* G6PD deficiency RDT kit among participants with known G6PD status (Normal and Deficient) from previous studies carried out in the study area. This study was conducted between February 2013 and June 2013.

To calculate the sample size to carry out the performance evaluation, the team assumed an expected Area under ROC curve of 0.80 compared to a Null hypothesis value of 0.68 [16] considering a 2:1 ratio of sample sizes in negative/positive groups at 95% CI with 80% power (MedCalc version 12.5 software, Mariakerke, Belgium). A minimum of 174 participants composed of G6PD Deficient (n = 58) and Normal (n = 116) was required to assess the diagnostic performance of the kit. The team increased the sample size by assuming a 20% refusal rate and the possibility of a prospective participant not available during recruitment in the field to generate the total listing that was sent to the field.

### Study site

**Fig 1:**
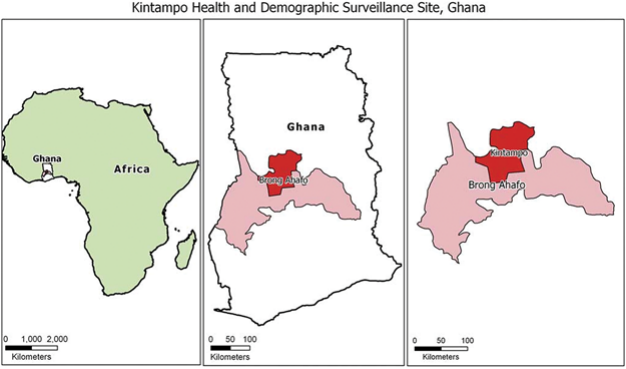
Map of the study communities.

The study was carried out in the Kintampo North Municipal and Kintampo South District of Ghana (Fig 1); two administrative areas where the population is periodically followed for health indicator data [10]. The study site covers an area of 7162 Sq Km with a resident population of approximately 140,000 [10]. The study area is located within the forest-savannah transitional ecological zone in Ghana and subsistent farming is the predominant occupation. The prevalence of malaria parasitemia is about 50% among children less than 10 years of age (symptomatic/asymptomatic) with perennial malaria transmission and entomological inoculation rate of 269 infective bites per person per year [7]. The prevalence of G6PD deficiency in the community is about 7% to 19% [1, 11]. There are more than twelve (12) clinics and three (3) hospitals within the study area. None of these health facilities performs the G6PD deficiency screening test as part of health service delivery except the research institution. Malaria rapid diagnostic test are however available at all health facilities and also used by community based health officers for malaria diagnosis.

### Study population, participants selection and recruitment

Kintampo Health Research Centre carried out a population reference study and also a two-year cohort study. G6PD deficiency testing was one of the laboratory investigations carried out in the two studies. The database of the population of the two studies became the sampling frame to select prospective participants to evaluate the diagnostic accuracy of the G6PD RDT kit.

The number of G6PD Deficient and Normal participants with known G6PD study participants with known G6PD status from the previous studies were selected from the database using MS Excel. The status of potential participants from the previous studies [21, 22] were determined using PCR [23, 24] and quantitative spectrophotometric method [25, 26].

**Fig 2:**
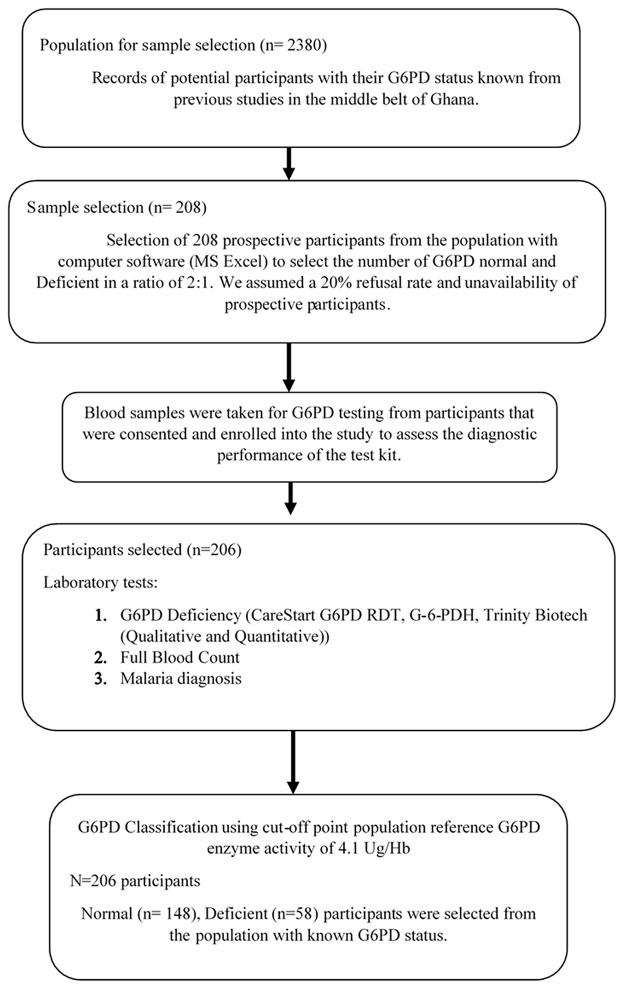
Flow chart of profile for study participants’ selection and recruitment.

Selected individuals were initially visited at home, informed about the study and invited to participate after consenting. Individuals who were present in the community and willingly agreed to be part of the study were invited to a central point within the community to interact with the study team. A prospective participant who refused consenting to be recruited was replaced from list of prospective participants with known G6PD status. Flow chart for study participants’ selection and recruitment is presented in Fig 2.

The process of participants’ selection was as follows. The team consulted a database from two previous studies that enrolled participants with their G6PD status established either with PCR or quantitative method. The team randomly selected the needed number of positive/deficient participants from the list of G6PD deficient individuals classified in the previous study. Same random selection was done to get the number of negative/normal individuals from the previous studies. Selected prospective participants from the previous studies were contacted in the community, consented to participate in the current evaluation study. The approach adopted in this study was not wholly community based survey and then presents the limitation of estimating prevalence parameters like the NPV and PPV. We can only describe the sensitivity, specificity and the Area under the ROC curve. We did not assign randomization codes or any special randomization technique. Simple randomization was adopted to fairly select any participant with known G6PD status in the category of interest.

### Participants’ sampling and data collection

G6PD deficiency test was done with *CareStart* G6PD deficiency RDT screening kit (Access Bio. Inc., New Jersey, USA, LOT No. GP3A1) using capillary blood samples collected from sterile finger prick. About 1.0–1.6ml of blood was collected after G6PD and malaria RDT testing from the finger prick. The capillary blood sample collected into the EDTA microtainer test-tubes were sent to the KHRC Clinical Laboratory to perform G6PD deficiency screening tests using Trinity Biotech quantitative G6PD assay (Cat. No. 345UV, Trinity Biotech, St. Louis, USA) and Trinity Biotech qualitative G6PD assay (Cat. No. 400K, Trinity Biotech, St. Louis, USA). Trinity Biotech quantitative method for G6PD was adapted and performed on The Flexor E Clinical Chemistry analyser (Vital Scientific, The Netherlands). Quantitative and qualitative analyses were performed on the fresh blood samples within a maximum of 48 hours after the collection to determine levels of G6PD enzyme activity.

### Laboratory test procedures

#### Quantitative determination of G6PD activity

Using Trinity quantitative method: The Trinity Biotech quantitative G6PD assay method was used as the gold standard [16]. The method is based on the spectrophotometric methods of Kornberg, 1955 [25] and Lohr et al, 1974 [26]. This involves the reduction of NADP to NADPH, by the G6PD enzyme in the presence of glucose-6-phosphate to the formation of NADPH which is proportional to the G6PD enzyme activity, measured spectrophotometrically at 340 nm (Table 1). The method is most accepted and sensitive and has been widely used to evaluate most G6PD methods.

**Table 1:** An outline comparing some common factors of importance between the methods used for G6PD screening in the study.

TEST NAME	TRINITY QUANTITATIVE	TRINITY QUALITATIVE	*CARESTART* G6PD RDT (QUALITATIVE)
**VOLUME OF SAMPLE NEEDED**	10μl*	5μl*	2.0μl
**APPROXIMATE TIME NEEDED TO READ RESULTS**	20mins	1hr:5min	10mins
TEMPERATURE AT WHICH TEST IS STABLE *(Manufacturer package)*	2°C–8°C	2°C–8°C	18°C–32°C
CAPABLE OF BEING USED FOR POINT-OF-CARE SCREENING	No	No	Yes

*: Volume of blood to estimate Haemoglobin concentration is needed.

#### Classification of G6PD deficiency

The classification of G6PD deficiency status, using quantitation assay methods were based on population reference value G6PD activity of adjusted male median estimated to be 5.5 U/g Hb ((Male Median = 5.5, Range (4.1–15.4)), *Unpublished data*) established in the study area using the classification procedure described by Domingo et al, 2013 [27]. The team classified and assessed the performance of the kit at enzyme activity levels <10% (n = 0), <20% (n = 1), <30% (n = 12), <40% (n = 14), <60% (n = 23) and above the median of normal G6PD enzyme activity recorded of participants used in this study. The population reference value G6PD activity was established using a population of 1,353 sampled randomly from the study area. The population sampling and recruitment for the data to establish this G6PD enzyme activity reference value are described in Dosoo et al, 2012 and Dosoo et al, 2014 [22, 28].

Using the Trinity Quantitative method, the G6PD deficiency status of participants were classified as either “Normal” or “Deficient” at a cut-off of 4.1 U/g Hb. All participants who measured G6PD enzyme activity <4.1 U/g Hb were classified as “Deficient” while those with enzyme activity equal to or above 4.1 U/g Hb were considered “Normal”.

#### Qualitative determination of G6PD activity and deficiency classification

**Fig 3:**
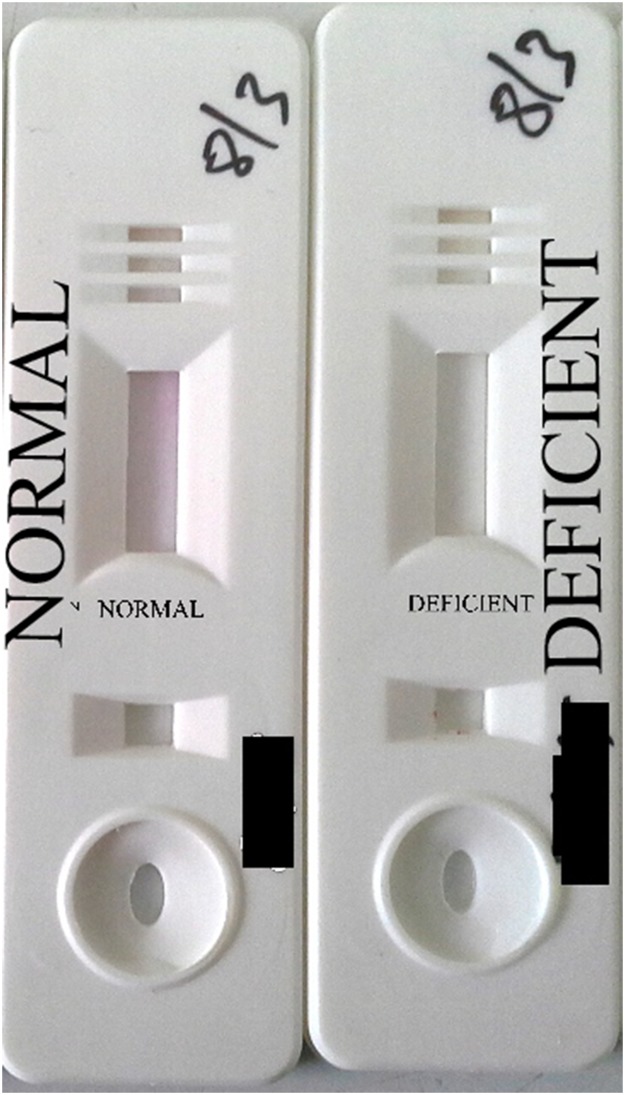
*CareStart* G6PD RDT screening kit with results interpretation. Results of kit labelled with NORMAL and DEFICIENT interpretation.

Using *CareStart* G6PD deficiency screening test: The *CareStart* G6PD deficiency RDT format test, which is a qualitative enzyme chromatographic test is based on the reduction of colourless nitro blue tetrazolium dye to dark coloured formazan. The test kit requires minimal training and no special expertise or additional equipment for use. Two microliters (2μl) of blood added into the sample well with two drops of buffer into the buffer well gave the G6PD deficiency status of an individual in 10 minutes. Samples with normal G6PD activity produce a distinct purple colour background in the result window while no colour change was observed for samples with deficient G6PD activity (Fig 3). Samples that produced a pale purple colour giving ambiguous *CareStart* test results were for safety reasons called deficient, which would be the norm in a clinical setting. This could result in a decrease in specificity. The performance of *CareStart* G6PD deficiency RDT screening kit was stable in a temperature of 20°C to 32°C *(was verified over a two-day period with Libero Ti1 digital thermometer*. *Data not shown)* (Table 1).

Using Trinity Qualitative method: The Trinity Biotech qualitative G6PD assay method uses G6PD released from lysed erythrocytes to catalyze the conversion of glucose-6-phosphate to 6-phosphogluconate with reduction of NADP to NADPH. In the presence of phenazine methosulfate (PMS), NADPH reduces a blue dye to the colourless form. The rate at which the colour visually disappears in the reaction mixture is proportional to the G6PD content of red cells. Disappearance of blue colour of the dye within 1 hour while incubating reaction mixture at 37°C denoted participant had NORMAL G6PD enzyme activity. DEFICIENT was when there was no change in the blue colour (Table 1).

#### Full blood count analysis

Full Blood Count was determined using a validated ABX Micros 60 analyzers (Horiba-ABX, Montpellier, France) to obtain the haemoglobin concentration, Red Blood Cells and White Blood Cell count levels of participants were used in the calculation of the G6PD activity in the erythrocytes and malaria parasite density.

### Malaria diagnosis

Malaria RDT test kits (*CareStart* Malaria RDT (Access Bio. Inc., New Jersey, USA)) were used to screen for malaria simultaneously with the G6PD RDT. Samples collected were transported to the KHRC laboratory to prepare blood slides to examine for malaria parasites. Microscopic examinations of thick and thin blood films were conducted to confirm malaria diagnosis. Thin and thick peripheral blood smears prepared were read by two independent microscopists, and by a third microscopist if the results were discordant on positivity or on parasite density, as described by Adu-Gyasi et al, 2012 [29].

### Data management and statistical analysis

Data was checked for completeness and consistency and all queries resolved after double data entry using Microsoft Access software 2010. Cleaned data was analysed using Stata Software version 12 (Stata Corporation, TX USA). The performance of G6PD deficiency RDT was expressed by calculating the sensitivity (SE), specificity (SP) and the Area under the Receiver Operator Characteristics (ROC) curve for G6PD deficiency status using the Trinity quantitative results as gold standard [16]. Differences in means and all reported parameters were considered significant at 95% Confidence Intervals.

### Quality Assurance

All 5 personnel involved in the various tests were trained before the start of the study. The training included classifying a qualitative test outcome as ‘‘positive” or ‘‘negative” (deficient or normal) as described by the manufacturer’s instructions. Qualitative tests were performed before the quantitative analysis. Personnel who performed the tests were blinded to the results of each method and stage of testing. Quality control checks were performed for the Full Blood Count on the automated haematology analyser with commercially available controls. The *CareStart* G6PD deficiency RDT and Trinity Quantitative methods were controlled with two levels of commercially prepared G6PD quality control (QC) (normal level, Cat. No. PD 2618 and deficient level Cat. No. PD 2617 controls) samples from Randox.

## Results

### Study population

A total of 206 participants were recruited. About 57.8% (119/206) of the participants were males and 42.2% (87/206) were females. The median age of the participants was 27 years (range 2–85 years); 21.4% (44/206) of participants were less than or equal to ten years.

### Comparing the performance of *CareStart* G6PD deficiency test method to the routinely used methods

**Fig 4:**
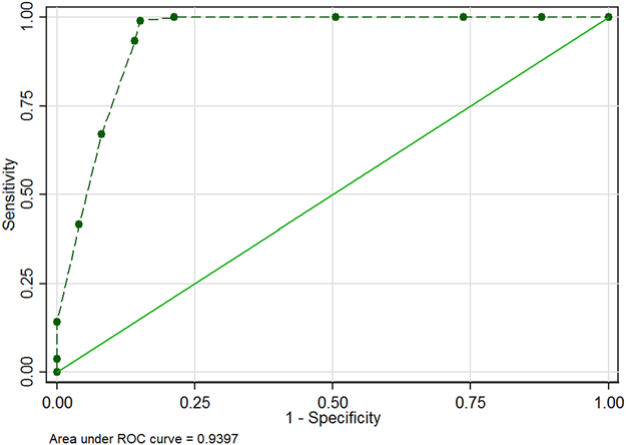
ROC curve of RDT performance with plotted points of considered enzyme activity.

G6PD deficiency RDT and Trinity Biotech quantitative: The SE and SP of *CareStart* G6PD deficiency RDT method were 100% and 72.1% using Trinity Biotech quantitative method as a gold standard. The ROC was 0.86 (Table 2). Considering the performance of the kit at (0.6, 1.1, 1.7, 2.2, 3.3, 4.4, and >4.4) Ug/Hb of G6PD enzyme activity, a ROC of 0.94 was obtained as presented in Fig 4. The sensitivity of the RDT kit was excellent at G6PD enzyme activity less than 8.0 Ug/Hb.

**Table 2:** Performance of the *CareStart* G6PD deficiency RDT kit to other *commercially available methods for screening G6PD deficiency*.

	Gold standard Deficient		Gold standard Normal				
	RDT Diff. (n)	RDTnormal (n)	RDT Diff. (n)	RDTnormal (n)	Sensitivity% (95% CI)	Specificity% (95% CI)	AUC(95% CI)
**Type of Gold standard method**							
Trinity Biotech Qualitative	90	1	4	102	98.9, (94.0, 100)	96.2 (90.6, 99.0)	0.98 (0.96, 1.00)
Trinity Biotech Quantitative	67	0	32	106	100 (94.7, 100)	72.1 (64.1, 79.2)	0.86 (0.83, 0.90)

G6PD deficiency RDT and Trinity Biotech qualitative: The SE and SP of *CareStart* G6PD deficiency RDT method were 98.9% and 96.2% comparing to Trinity Biotech Qualitative method. The ROC was 0.98 (Table 2). When the Trinity biotech qualitative method was compared to the Trinity Biotech quantitative method as “gold standard” the SE and SP were 100% and 76.3% with a ROC of 0.88.

### Comparing the performance of *CareStart* G6PD RDT kit by gender to routinely used methods to screen for G6PD deficiency.

The diagnostic accuracy of *CareStart* G6PD RDT to screen for G6PD deficiency when compared to Trinity biotech qualitative (P = 0.124) and Trinity biotech quantitative (P = 0.123) method were not significantly affected by gender (Table 3).

**Table 3:** Diagnostic performance of *CareStart* G6PD RDT by gender compared to Trinity Biotech Quantitative and Qualitative methods for G6PD deficiency screening.

		Gold standard Deficient		Gold standard Normal				
		RDT Diff. (n)	RDT normal (n)	RDT Diff. (n)	RDT normal (n)	Sensitivity % (95% CI)	Specificity % (95% CI)	AUC (95% CI)
Sex	**Type of Gold standard method**							
Males	Trinity Biotech Qualitative	57	0	1	58	100 (93.7, 100)	98.3 (90.9, 100)	0.99 (0.97, 1.00)
	Trinity Biotech Quantitative	50	0	10	59	100 (92.9, 100)	85.5 (75.0, 92.8)	0.93 (0.89, 0.97)
Females	Trinity Biotech Qualitative	33	1	3	44	97.1 (84.7, 99.9)	93.6 (82.5, 98.7)	0.95 (0.91, 1.00)
	Trinity Biotech Quantitative	17	0	22	47	100 (80.5, 100)	68.1 (55.8, 78.8)	0.84 (0.79, 0.90)

### Performance of *CareStart* G6PD deficiency RDT Kit among malaria participants

**Fig 5:**
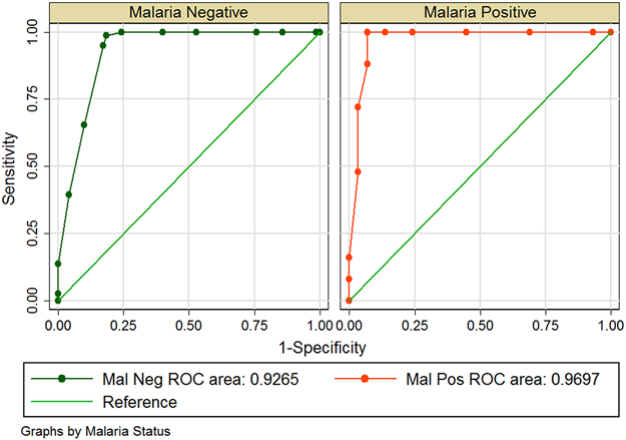
Area under the Receiver Operator Characteristics (ROC) curve for G6PD RDT performance considering malaria status of participants.

**Fig 6:**
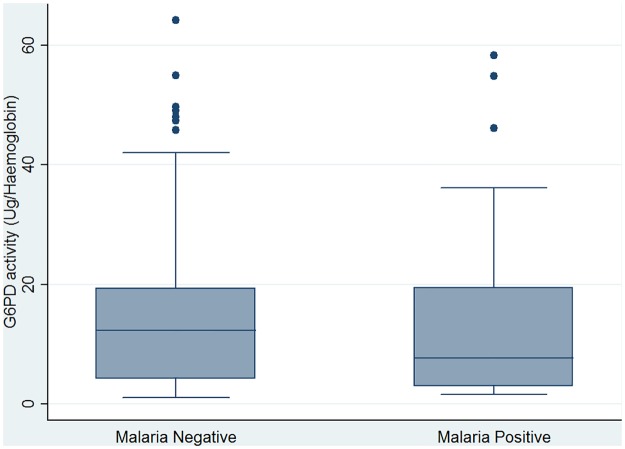
Estimates of G6PD enzyme activity levels by malaria parasite status.

Among the study participants, 26.2% (54/206) had positive malaria parasites using microscopy. The performance of *CareStart* G6PD deficiency RDT was not affected significantly (P = 0.199) when we considered participants that had malaria parasites infection compared to those that had no malaria parasite (Fig 5). The difference in the Mean (±SE) of levels of G6PD enzyme activity among participants with positive malaria parasites (95% CI, 13.0 (±1.81)) compared to those with negative malaria parasites (95% CI, 14.4 (±1.0)) was not significant (P = 0.242). Fig 6, illustrates the distribution of the levels of G6PD enzyme activity among participants considering malaria parasite status.

## Discussion

Early diagnosis of Glucose-6-phosphate dehydrogenase deficiency could avert the potentially harmful effect of the deficiency encountered during patient management with primaquine [30, 31] which is being recommended to be administered at fixed dose with ACT for malaria elimination in endemic regions [15, 31]. The danger of treating patients with drugs that are contraindicated for individuals with this deficiency could range from mild to severe haemolysis.

Ghana does not use primaquine in the management of patients with malaria [12] since it is mainly employed in treating patients with relapsing *Plasmodium species* infections. Adoption of the WHO recommendation would mean deploying treatment with primaquine and ACTs in most malaria endemic regions including Ghana especially with the target of eliminating *Plasmodium falciparum* gametocytes[6, 15]. Implementation of the recommendation of primaquine fixed dose by WHO faced a challenge of seldom availability of G6PD testing in the field [31]. The *CareStart* G6PD RDT which has high and acceptable diagnostic performance as assessed in this study could make G6PD testing available at point-of-care and alleviate this challenge.

The SE and SP of the *CareStart* G6PD deficiency RDT compared to results from Trinity quantitative method used as the “gold standard” in a high malaria endemic area in Africa was higher than what was recorded in the evaluation study by Kim et al, 2011 [16]. This diagnostic performance of the kit was acceptable and comparable to performance documented in other studies that evaluated the G6PD RDT kit [17, 18, 20, 32]. It was evident from this study that the test kit has the highest sensitivity at concentrations of G6PD enzyme activity less than 8 Ug/Hb. This performance of the test kit successful in detecting individuals with lower G6PD enzyme activity is comparable to what von Fricken et al, 2014 and Roca-Feltrer et al, 2014 [17, 18] established which confirms its suitability [18, 19] as point-of-care device to screen for G6PD deficiency before administration of any drug that can induce acute haemolysis. The performance of the test kit was similar among participants with malaria parasites and those without parasites using microscopy (Fig 5). This assuring information also presents the device as suitable to facilitate the testing for G6PD deficiency before drugs are administered during malaria patients’ management in peripheral health facilities where laboratory services are absent [18, 20].

Similarly, the BinaxNow qualitative method has a good performance but the range of temperatures (18°C to 25°C) [32] required to carry out this test could compromise on its use for point-of-care testing in a resource deprived facility in the tropics where temperature may rise up to 32°C [33]. It is also overly clear that in a resource constraint facility, other highly performing rapid G6PD screening methods such as the Fluorescent Spot Test cannot be used since additional equipment requirements and specified storage conditions are needed for the testing processes making the methods expensive to be carried out [18–20].

Obviously, most of the methods available and accepted for routine screening of G6PD status of patients are not suitable to be employed to screen for G6PD status of patients at point-of-care in malaria endemic regions in the tropics where screening for G6PD is most needed. These rapid methods need sophisticated equipment and temperature regulation. Again, unlike the available routine test methods that require a minimum of 5μl to 3000μl of blood depending on the method to use, this screening kit requires a volume of 2ul of capillary blood to determine the G6PD deficiency status of an individual in 10mins at a temperature range of 18°C to 32°C (Table 1). This also makes the device acceptable for use among paediatric patients where large volumes of samples needed for testing are matters of concern.

### Limitations

None of the test cassettes had control window (*Manufacturer’s manual)*. It would have been preferable to have this in the design to control each test that is performed with the kit. Analyzing each batch of samples with controls might not be cost effective. Also, the *CareStart* G6PD deficiency RDT kit produce a purple colour to differentiate between normal and deficient subjects (*Manufacturer’s manual*). Borderline results also produce a pale purple colour. This can result in operator-to-operator variations due to the subjectivity of interpretation for qualitative colormetric results. The development of a technique to semi-quantitate the results of the rapid test would be helpful to minimise misinterpretation of RDT results as mentioned by others.

The study participants were selected from a pre-selected population with known G6PD status. This presents the limitation of estimating prevalence parameters like the negative predictive values (NPV) and positive predictive values (PPV). The team could only describe the sensitivity, specificity and the Area under the ROC curve to assess the diagnostic performance of the kit.

### Conclusion

The results from this study suggest that the diagnostic performance of the *CareStart* G6PD RDT is acceptable at determining the G6PD deficiency status in a high malaria endemic area in Ghana, Africa. The *CareStart* G6PD RDT kit presents as an attractive tool for point-of-care G6PD deficiency testing in areas with high temperatures and requires significantly less time and training to implement. The G6PD RDT kit could be used to screen malaria patients before administration of the fixed dose primaquine with ACT as recommended by the WHO.
